# GJB3: a comprehensive biomarker in pan-cancer prognosis and immunotherapy prediction

**DOI:** 10.18632/aging.205774

**Published:** 2024-05-07

**Authors:** Jingtong Zeng, Xianjie Li, Yifan Zhang, Bo Zhang, Hanqing Wang, Shihao Bao, Lingling Zu, Hao Zhang, Yuan Cheng, Quanying Tang, Xiaohong Xu, Song Xu, Zuoqing Song

**Affiliations:** 1 Department of Lung Cancer Surgery, Tianjin Medical University General Hospital, Tianjin, China; 2 Tianjin Key Laboratory of Lung Cancer Metastasis and Tumor Microenvironment, Lung Cancer Institute, Tianjin Medical University General Hospital, Tianjin, China; 3 Colleges of Nursing, Tianjin Medical University, Tianjin, China

**Keywords:** GJB3 (Cx31) gene, pan-cancer, connexin, TCGA, LUAD

## Abstract

Background: A wide range of connexins are situated between normal-normal cells, cancer-cancer cells, and cancer-normal cells. Abnormalities in connexin expression are typically accompanied by cancer development; however, no systematic studies have examined the role of Gap Junction Protein Beta 3 (GJB3) in the context of tumor progression and immunity, especially when considering a broad range of cancer types.

Methods: In this study, data on GJB3 expression were gathered from Genotype-Tissue Expression, Cancer Cell Line Encyclopedia, and The Cancer Genome Atlas databases. Then, we analyzed the relationship between GJB3 expression and tumor characteristics. *In vitro* experiments using colony formation, EdU, CCK8, transwell migration assays, immunohistochemistry and western blot were performed to investigate the function of GJB3 in tumor progression of various cell lines. A drug sensitivity analysis of GJB3 was performed using the Genomics of Drug Sensitivity in Cancer database.

Result: Our findings demonstrate that GJB3 is widely expressed in various cancers and correlates significantly with disease stages, patient survival, immunotherapy response, and pharmaceutical guidance. Additionally, GJB3 plays a role in different cancer pathways, as well as in different immune and molecular subtypes of cancer. Co-expression of GJB3 with immune checkpoint genes was observed. Further experiments showed that knockdown of GJB3 inhibited the PI3K/AKT pathway and resulted in reduced proliferation, migration, and viability of different cancer cells.

Conclusion: Overall, GJB3 shows potential as a molecular biomarker and therapeutic target for various cancers, particularly lung adenocarcinomas, mesothelioma, pancreatic adenocarcinoma. Thus, GJB3 may represent a new therapeutic target for a wide range of cancers.

## INTRODUCTION

Malignant tumors have always been a threat to human health, and remain a leading cause of death worldwide. Among all cancers, breast cancer, lung cancer, and colorectal cancer are highly prevalent and globally acknowledged as having the highest mortality rates [1, 2]. In humans, the suppression and elimination of malignant tumors is a perennial issue; however, due to the complexity of malignant tumors, the associated clinical outcome is generally unfavorable [3]. This is attributed in large part to the absence of effective therapeutic targets to treat these tumors.

The connexin family is comprised of at least 20 homologous proteins in the human body. These proteins form aqueous channels that connect the interiors of coupled cells and act as a communication medium for electrical and chemical signals [4]. Generally, connexins are considered to be tumor suppressive; however, recent studies using clinical samples indicate that connexins play a different role depending on the type of tumor. For example, connexin 30 expression inhibits the growth of malignant gliomas, but is protective against radiation treatment [5]. Furthermore, it has been observed that connexin 30.3 can enhance gastric cancer cell proliferation and migration through activation of the Wnt/CTNNB1 pathway [6].

According to these findings, the function and role of the connexin protein in various types of cancer is variable. Although there have been a large number of studies conducted on members of the connexin family to date, connexin 31, also known as Gap Junction Protein Beta 3 (GJB3), has not received much attention, especially from a pan-cancer perspective. This study provides a comprehensive investigation of the GJB3 gene expression signature and its prognostic significance across multiple human cancers. And also evaluated the relevance of GJB3 expression on immune checkpoint-related genes and immune cell infiltration scores. In addition, the role of GJB3 in the efficacy of immunotherapy was discussed. In summary, the present research demonstrated that GJB3 may play a role in the development of several different types of cancer, especially Lung adenocarcinoma (LUAD), Pancreatic adenocarcinoma (PAAD) and Mesothelioma (MESO). It has the potential to serve as an efficient biomarker for predicting the clinical outcome and effectiveness of immune therapy.

## MATERIALS AND METHODS

### Expression levels of GJB3 in various sources

Research was conducted on the abnormal expression of GJB3 in different cancers and healthy tissues by integrating the Genotype-Tissue Expression (GTEx) portal for normal tissue data with The Cancer Genome Atlas (TCGA) database for cancer data. Analyses of GJB3 expression in a variety of tumor cell lines were conducted using the Cancer Cell Line Encyclopedia (CCLE) portal [7]. In this study, 33 cancer types were examined (Supplementary Table 1). RNAseq data from the TCGA and GTEx in FPKM format was used to ensure comparison of uniform expression data. The transcriptome data for all disease samples have been processed and standardized as log2(FPKM + 1).

### Prognostic analysis

Using the R “Survival” package, Kaplan-Meier (KM) method and univariate Cox proportional hazards models were constructed. Using the above methods, investigate whether GJB3 plays a role in cancer prognosis, including overall survival (OS), disease-specific survival (DSS), and progression-free interval (PFI). To visualize the hazard ratio (HR) and the corresponding *p*-values with their 95% confidence intervals, the forestplot R package was used to perform the univariate Cox analysis.

### Infiltration analysis of the immune system

The CIBERSORT algorithm and TIMER algorithm, which are capable of predicting the phenotype of immune cells, were used to calculate the relative score for immune cells in cancers [8, 9]. The level of immune scores was calculated for each sample based on the gene expression signatures.

### Predictive analysis of immunotherapy effects

Based on Pearson correlation coefficients, the expression of GJB3 was examined in relation to a variety of immune checkpoint-related genes. These genes included PDCD1, CTLA4, LAG3, TIGIT and etc. Biologically, tumour mutational burden (TMB) refers to a marker that indicates the proportion of tumor cells containing somatic mutations [10]. DNA mismatch repair deficiency causes microsatellite instability (MSI), an indicator of anti-PD-1/PD-L1 immunotherapy efficacy. The MSI data were derived from previously published research [11–13]. Both the TMB and MSI can indicate the effectiveness of immunotherapy in treating tumors. Based on TMB and MSI, we explored the association between GJB3 and immunotherapy efficacy prediction, and the findings were illustrated using radar plots.

### Enrichment analysis

The R package “limma” was used to analyze gene expression differences between the samples of cancer and control. A Kyoto Encyclopedia of Genes and Genomes (KEGG) pathway enrichment analysis was used to examine the biological and molecular functions of GJB3 in various cancers. The KEGG dataset was analyzed in the “ClusterProfiler” package and the “enrichplot” package within the “R studio” platform.

### Cell lines, cell culture, and transfection

The LUAD cell lines (H2030, DV90), PAAD cell line (PANC1), and MESO cell line (H2452) were kindly provided by the American Type Culture Collection (ATCC). All the cells were maintained in a humidified atmosphere at 37°C with 5% CO_2_/95% air and cultured in RPMI 1640 or DMEM medium (Gibco, NY, USA) supplemented with 10% fetal bovine serum (Gibco, NY, USA). Small interfering RNA (siRNA) tagged GJB3, was purchased from Integrated Biotech Solutions (Shanghai, China). DV90 and H2030 cells were transfected with siRNA using Lipofectamine 3000 (Invitrogen, CA, USA) according to the manufacturer’s instructions. RT-qPCR was used to detect silencing efficiency of Small interfering RNA. The siRNA sequences are listed in Supplementary Table 2.

### Drug sensitivity and GJB3 expression analysis

Genomics of Drug Sensitivity in Cancer (GDSC) database was used to collect drug sensitivity data [14]. The level of GJB3 expression in various cell lines was obtained from CCLE. A Spearman correlation analysis was performed to investigate the significance of gene expression in relation to the drug response. IC50 represents the half maximal inhibitory concentration, the concentration required to inhibit tumor cell growth by 50%. The IC50 value indicates the sensitivity of cells to drugs, and the value is the concentration at which half of the cells are inhibited from growing. It is generally believed that drugs are more effective when their IC50 value is lower, indicating that they need a lower concentration to inhibit cell growth. Spearman correlation coefficients were used to examine the interrelationship between the IC50 values of each compound with the level of GJB3 expression in various tumor cell lines.

### RNA extraction and real-time polymerase chain reaction (RT-PCR)

The total RNA from the cells was extracted using TRIzol reagent (Invitrogen). Next, cDNA was synthesized with Prime Script RT Master Mix (TaKaRa, Dalian, China). The expression of target genes was analyzed using quantitative real-time PCR (qRT-PCR) with SYBR Green Master Mix (Applied Biosystems, MA, USA). The relative expression of target genes was normalized to GAPDH expression by using the 2^−ΔΔCT^ method to quantify relative gene expression. Detailed primer sequences are presented in Supplementary Table 2.

### Colony formation assays

Cell proliferation ability was measured using a plate colony formation assay. Briefly, 500 cells were seeded into each well of a six-well plate and incubated for approximately two weeks until a colony was obviously formed, and the medium was changed regularly. Next, the plate was gently washed with phosphate-buffered saline (PBS) and stained with 0.1% crystal violet, after which the number of colonies was counted.

### Assays for cell counting kit-8

Cell proliferation was assessed using a cell counting kit-8 (CCK8) assay. The 3000 cells were seeded in a 6-well cell culture plate, and incubated for 1, 2, 3, 4, and 5 days. 20 μL cell counting kit-8 solution was added at the same time each day, and the cells were maintained at 37°C for 2 h. In the following step, cell proliferation capacity was measured through absorbance at 450 nm using a microplate spectrophotometer (BioTek, Instruments, Inc., Winooski, VT, USA).

### Migration and invasion assays

To assess cell migration and invasiveness, 24-well Transwell inserts with 8-micron pore sizes (Corning Inc., USA) with or without Matrigel (Corning Inc., USA) were utilized. For the invasion assay, cells were seeded in the upper chambers coated with Matrigel and allowed to invade into the lower chambers containing 20% FBS. After 24 hours, the cells that invaded the Matrigel were fixed, stained and counted. For the migration assay, cells were seeded into the upper chamber without Matrigel and allowed to migrate into the lower chamber containing 20% FBS. Both assays were performed in triplicate and the number of cells was counted in 3 random fields.

### Western blotting

Total proteins were lysed with an appropriate amount of RIPA lysate (Beyotime, #P0013C, China) for 30 min, centrifuged at 12,000 r/min at 4°C for 10 min, and the supernatant was collected. The equivalent amounts of protein were separated by 10% SDS-PAGE and transferred to polyvinylidene difluoride (PVDF) membranes (Millipore, IEVH00005). Primary antibodies used were AKT (Cell Signaling, #4691, 1:1000), PI3K (Cell Signaling, #4292, 1:1000), Phospho-AKT (Cell Signaling, #4060, 1:1000), phospho-PI3K (Cell Signaling, 17366, 1:1000), GAPDH (Proteintech, #10494-1-AP, 1:50000) and GJB3 (Proteintech, #10494-1-AP, 1:1000). Secondary antibodies used were HRP-conjugated anti-mouse and anti-rabbit IgG (Abclonal, #AS014, 1:10000). Protein bands were visualized by using ECL detection reagents (Millipore, WBULS0500).

### Immunohistochemistry

Tissue samples from multiple tumor tissue microarrays were used for clinical validation, containing the tumor and adjacent normal tissues from Melanoma. Additionally, six paired frozen fresh tumor tissues and adjacent non-tumor lung tissues were collected from patients with lung adenocarcinoma at Tianjin Medical University General Hospital (Tianjin, China). All samples were collected with informed consent from the patients. All the related procedures were performed with the approval of the Internal Review and Ethics Committee of Tianjin Medical University General Hospital. The sections were placed in an oven at 56°C for 1 h to melt the paraffin and prevent tissue shedding before dewaxing. The next step was to remove the paraffin with xylene and alcohol, and then boil them in sodium citrate buffer (pH 6.0) for antigen retrieval. The primary antibody GJB3 (Proteintech, #10494-1-AP,1:200) was added and incubated overnight at 4°C in a humidified box. The next day, the primary antibody was washed away with PBS, the secondary antibody was added, and these sections were incubated at 37°C for 40 minutes. After washing off the secondary antibody with PBS, it was developed with diaminobenzidine tetrachloride (DAB), and the nucleus was stained with hematoxylin. Finally, we covered the sections with neutral balsam and coverslips. In addition, two pathologists were consulted to ensure the typicality of the selected tissues.

### Edu assay

The different groups of cells were seeded into a confocal dish at a density of 5 × 10^5^ cells/200 μL. Paraformaldehyde (4%) was used to fix the cell for 10 min. After washing with PBS, the triton (1%) was used to transparent the cell for 5 min. According to the manufacturer’s manual, the cells were incubated with dyeing agent for 30 min in the dark, stained with Azide 488/555 and incubated for 5 min at 37°C. After washing twice with PBS containing Tween-20 (PBST), the images were captured using a fluorescence microscope (Olympus BX51, Japan). The reagents used in the EDU assay were purchased from Beyotime Biotechnology (C0075S, Beyotime, China).

### Statistical analysis

Statistical analysis of all data in this study was performed using R software version 4.2.2, and GraphPad Prism 8 was utilized for further statistical analysis. Statistical analyses, including *t*-tests and Wilcoxon rank sum tests, were employed to compare data between groups and determine statistical significance. A Pearson’s product-moment correlation coefficient was utilized to assess the correlation between two variables. The threshold of statistical significance was set to *P* < 0.05.

### Data availability statement

The data analyzed in this study can be found in online repositories. The names of the repositories and accession numbers are included in the article.

## RESULTS

### GJB3 expression in pan-cancer and normal tissues

**Figure 1:**
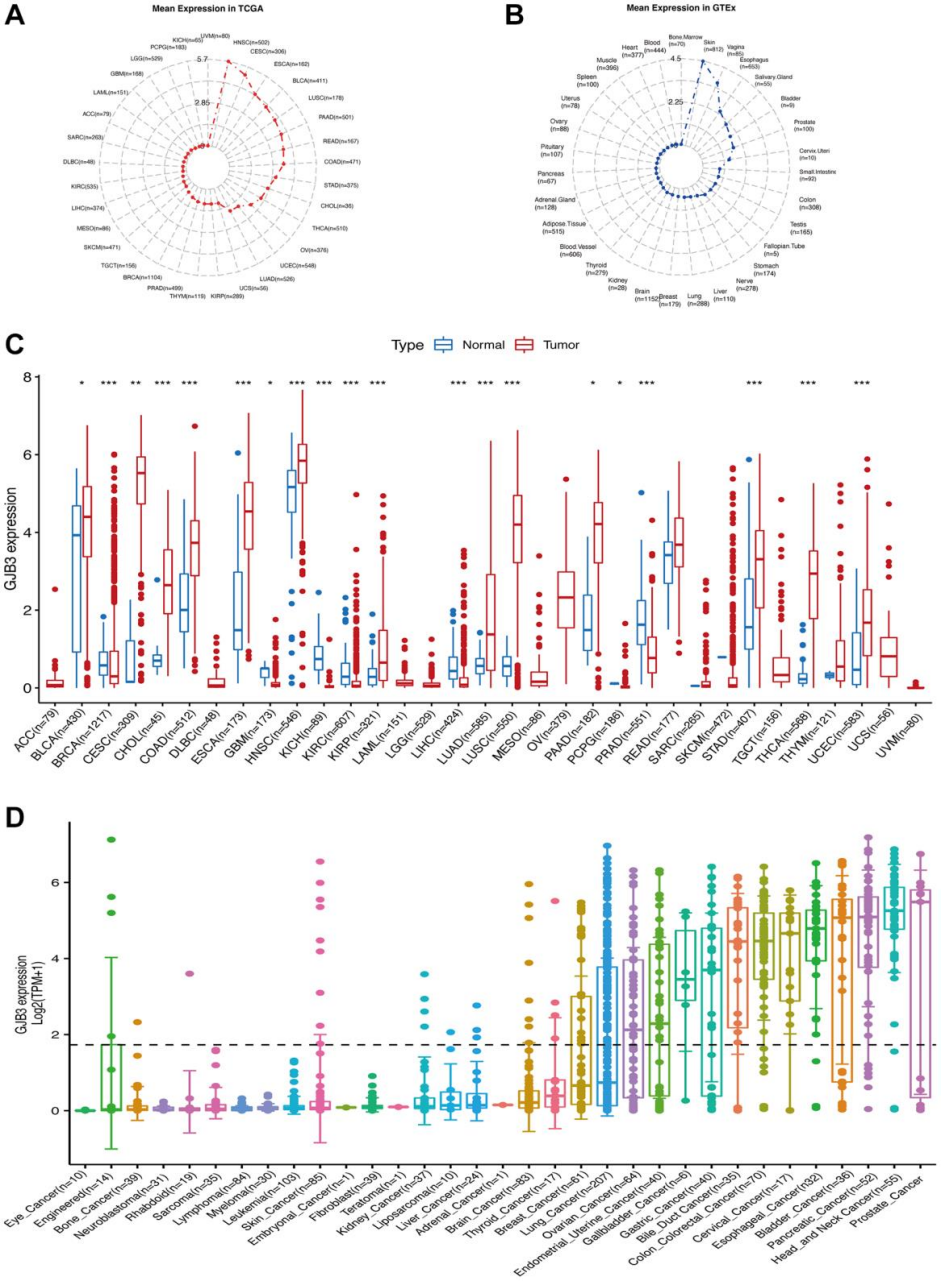
**The level of GJB3 mRNA expression was assessed in a variety of human cancers and normal tissues.** (**A**) The level of GJB3 expression in tumor tissues from TCGA database. (**B**) Level of GJB3 expression in normal tissues from the GTEx database. The dots represent the mean value of GJB3 expression. (**C**) Analysis of GJB3 expression in matched tumor tissues and normal tissues using data from the TCGA database. (**D**) The levels of GJB3 expression in tumor cell lines from the CCLE database (^*^P < 0.05; ^**^P < 0.01; ^***^P < 0.001; ^****^P < 0.0001).

As a first step, we performed expression analyses of the GJB3 gene in 33/31 types of tumor-normal tissue derived from TCGA and GTEx databases, to determine its level of expression. The results indicated that the level of GJB3 expression was highest in HNSC, and lowest in UVM in various tumors (Figure 1A). The skin was the tissue with the highest GJB3 expression, and the bone marrow exhibited the lowest level of expression (Figure 1B). Our study revealed that GJB3 expression was increased in the majority of types of cancer, such as Bladder urothelial carcinoma (BLCA), Cervical squamous cell carcinoma and endocervical adenocarcinoma (CESC), Cholangiocarcinoma (CHOL), Colon adenocarcinoma (COAD), Esophageal carcinoma (ESCA), Head and neck squamous cell carcinoma (HNSC), Kidney renal papillary cell carcinoma (KIRP), LUAD, Lung squamous cell carcinoma (LUSC), PAAD, Stomach adenocarcinoma (STAD), Thyroid carcinoma (THCA), and Uterine corpus endometrial carcinoma (UCEC, Figure 1C). Moreover, the abundance of GJB3 expression was significantly increased in different cancer cell lines sourced from the CCLE database, and was also an expression trend in various types of cancers, which were similar to that of previous findings (Figure 1D).

### GJB3 expression is considered to be a prognostic factor

**Figure 2:**
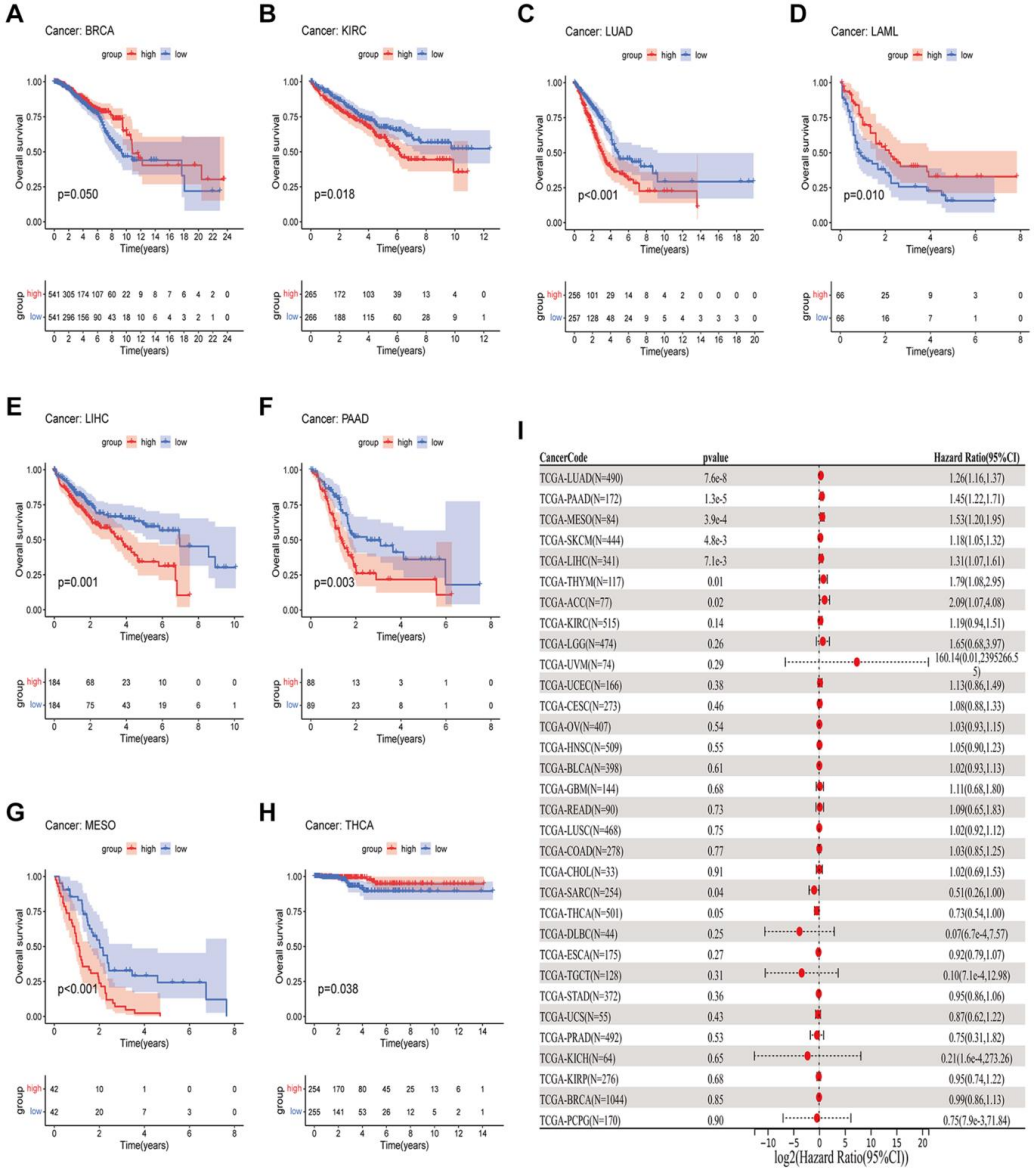
**A comparison of OS prognosis in various types of cancers according to GJB3 expression.** (**A**–**H**) In the Kaplan-Meier curves, GJB3 expression is correlated with OS expression. (**I**) Forest plot demonstrating the association between GJB3 and OS.

**Figure 3:**
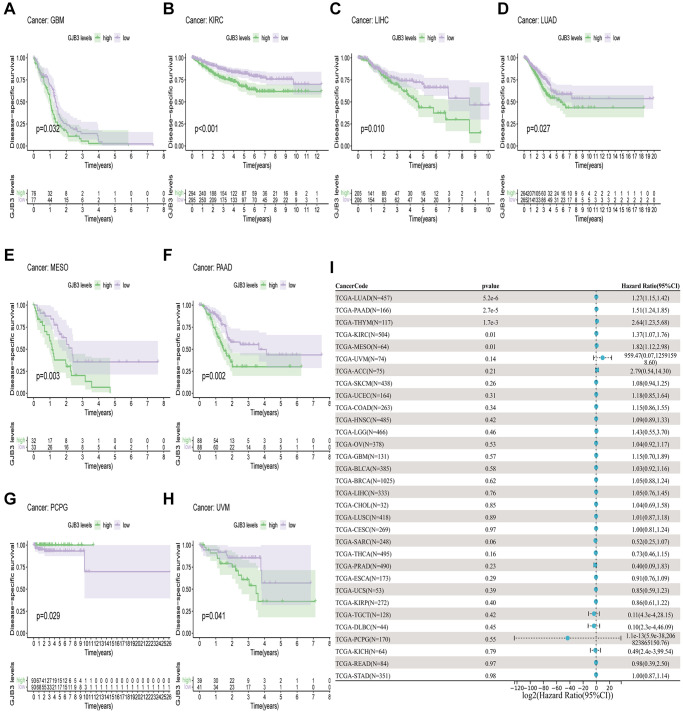
**A comparison of DSS prognosis in various types of cancers according to GJB3 expression.** (**A**–**H**) In the Kaplan-Meier curves, GJB3 expression is correlated with DSS expression. (**I**) Forest plot demonstrating the association between GJB3 and DSS.

**Figure 4:**
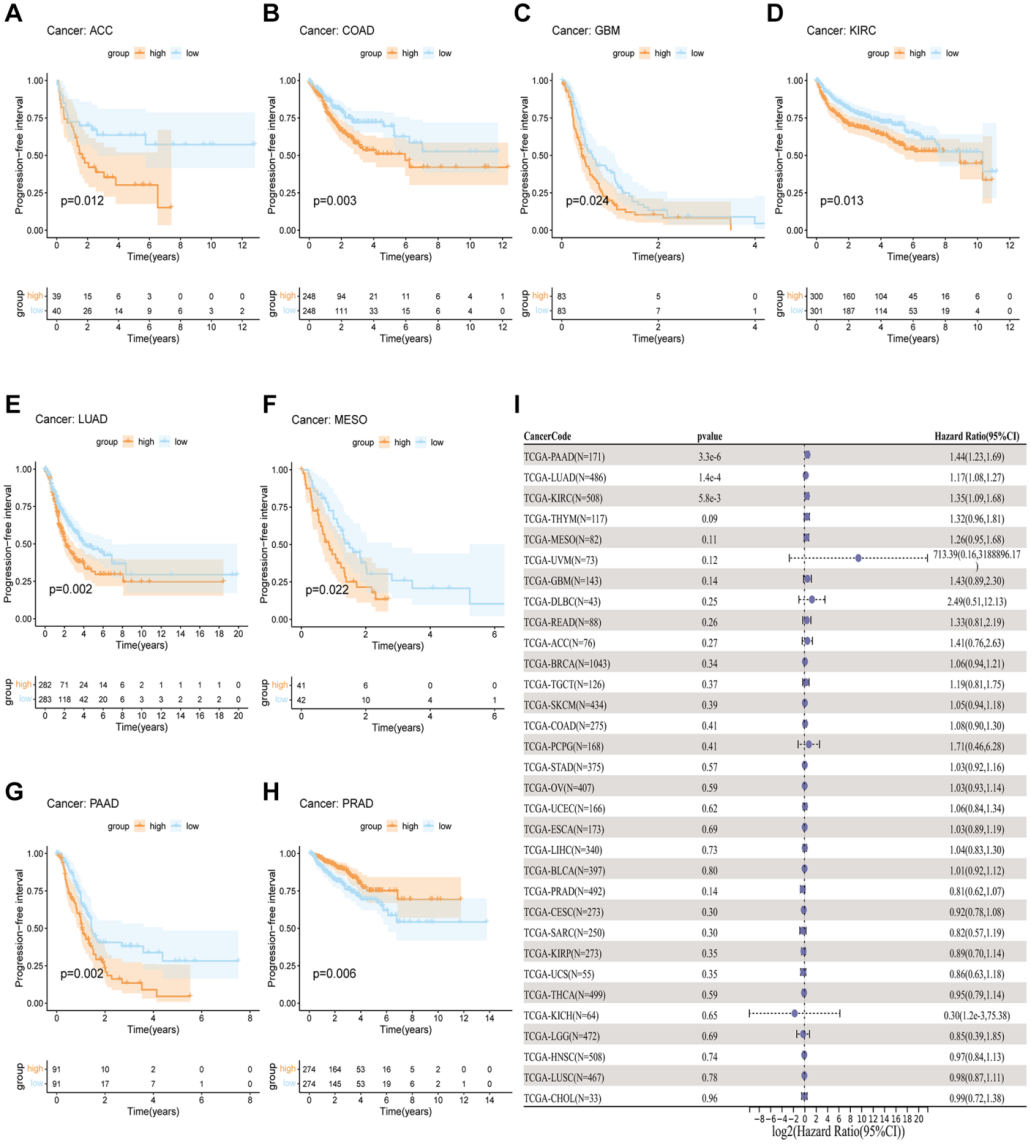
**A comparison of PFI prognosis in various types of cancers according to GJB3 expression.** (**A**–**H**) In the Kaplan-Meier curves, GJB3 expression is correlated with PFI expression. (**I**) Forest plot demonstrating the association between GJB3 and PFI.

In order to measure the survival significance of GJB3, a survival analysis was conducted utilizing TCGA data. Based on the KM survival curve results, we found that the high expression of GJB3 in Kidney renal clear cell carcinoma (KIRC), LUAD, Liver hepatocellular carcinoma (LIHC), PAAD, and MESO was associated with a shorter OS. In contrast, patients with higher GJB3 levels had a longer OS in Breast invasive carcinoma (BRCA), Acute myeloid leukemia (LAML), and THCA (Figure 2A–2H). As revealed by the Cox regression analysis, high GJB3 expression was associated with a shorter OS for LUAD, PAAD, MESO, Skin cutaneous melanoma (SKCM), LIHC, Thymoma (THYM), and Adrenocortical carcinoma (ACC, Figure 2I). As well, we examined the association between patients’ DSS and PFI and the level of GJB3 expression. The KM analysis also demonstrated that high GJB3 expression was associated with a worse DSS (Figure 3A–3H) and PFI (Figure 4A–4H) of patients with Glioblastoma multiforme (GBM), KIRC, LUAD, MESO, and PAAD. Furthermore, the Cox regression analysis demonstrated that the level of GJB3 in LUAD, PAAD, THYM, KIRC, and MESO has been identified as a risk factor for both DSS (Figure 3I) and PFI (Figure 4I) in patients. As a consequence of the findings of our study, it appears that GJB3 seems to be significantly related to the prognosis of multiple types of cancer; in particular, LUAD, PAAD, and MESO were significantly associated with patient OS, DSS, and PFI.

### GJB3 is an indicator of clinical stage

**Figure 5:**
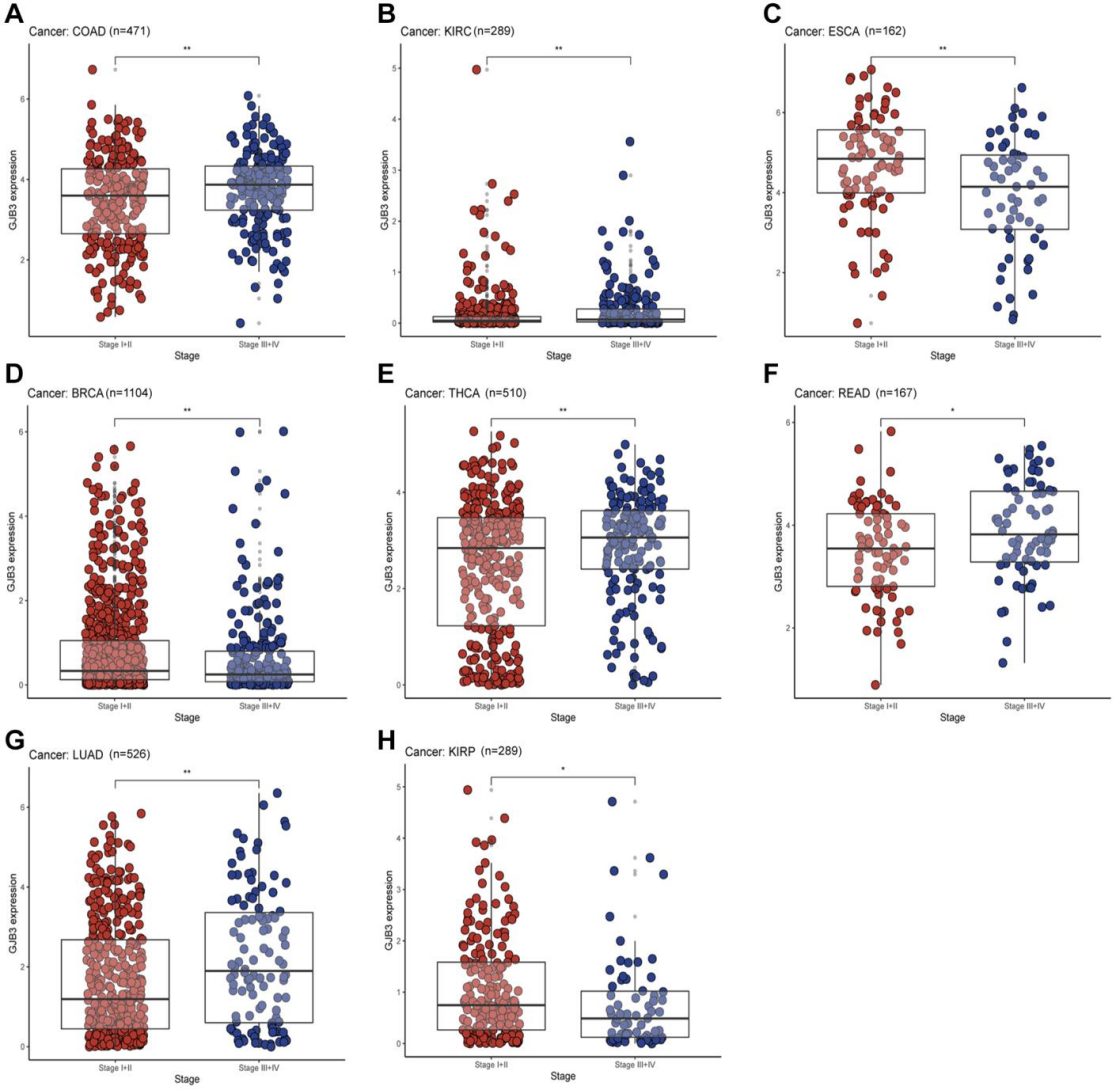
**With different cancers, GJB3 expression varies depending on clinical characteristics.** (**A**–**H**) Analysis of the relationship between GJB3 level and clinicopathological stage in COAD, KIRC, ESCA, BRCA, THCA, READ, LUAD, and KIRP patients. (^*^P < 0.05; ^**^P < 0.01; ^***^P < 0.001; ^****^P < 0.0001).

To further explore the differences between GJB3 at different stages of clinical and pathological development. Based on the tumor clinicopathological stage, we evaluated the level of GJB3 expression in early (Stages I and II) and advanced tumors (Stages III and IV). Additionally, based on clinicopathological features, the findings show that high GJB3 expression represents a higher degree of malignancy in COAD, KIRC, Rectum adenocarcinoma (READ), and LUAD. But in ESCA, BRCA, and KIRP cancers, the advanced patients expressed significantly less GJB3 (Figure 5A–5H). Collectively, these findings suggest that GJB3 may have both tumor suppressive and tumor promoting properties. There are therefore promising implications for the use of GJB3 as a biomarker in the detection, staging, and monitoring of cancers.

### The immunological properties of GJB3

**Figure 6:**
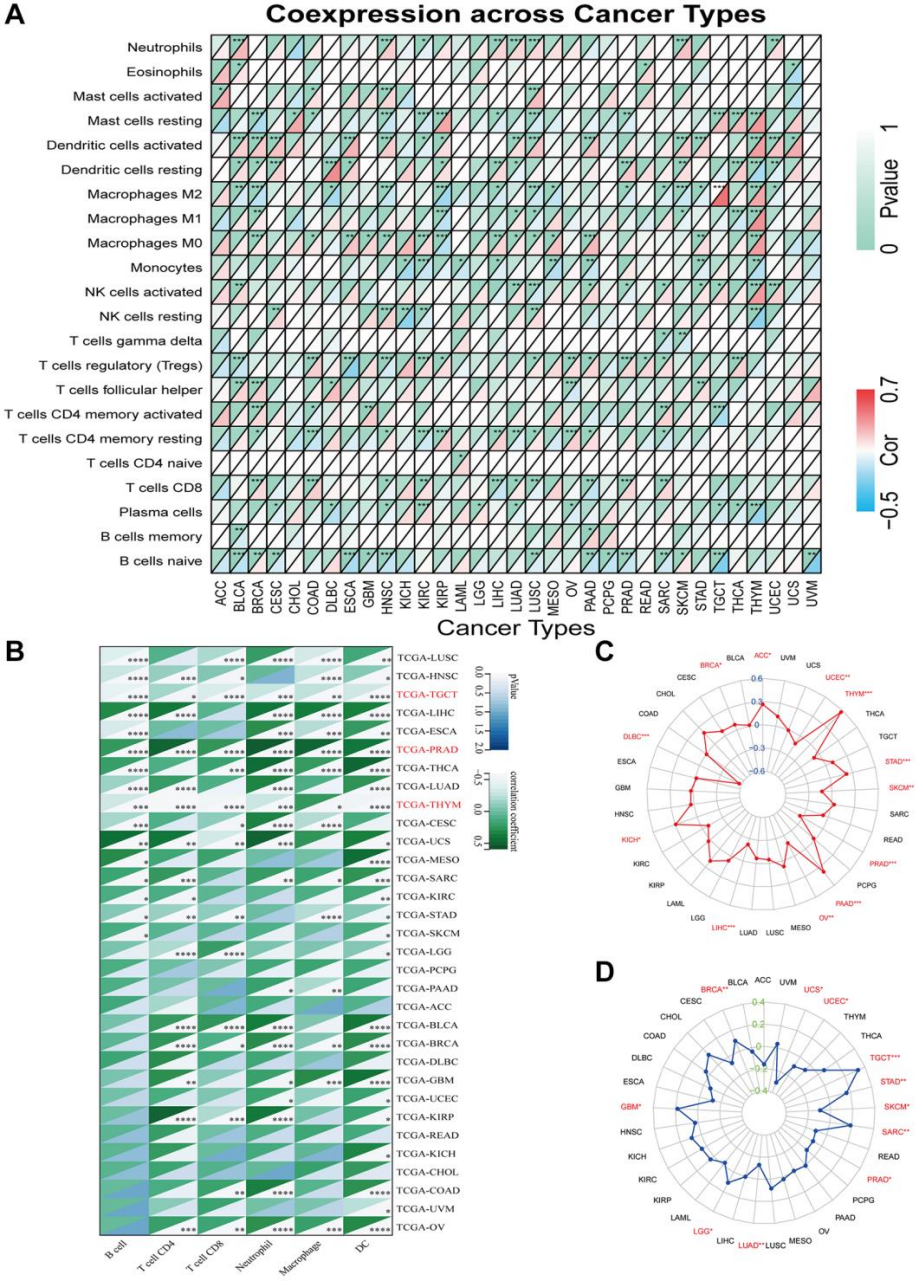
**Immune characteristics related to GJB3 expression.** (**A**, **B**) This heatmap displays a relationship between GJB3 level and the immune cell infiltration value calculated by the CIBERSORT (**A**) and TIMER algorithms (**B**) among pan-cancer specimens. (**C**, **D**) The correlation between GJB3 expression with TMB (**C**) and MSI (**D**) in multiple cancers. (^*^P < 0.05; ^**^P < 0.01; ^***^P < 0.001; ^****^P < 0.0001).

**Figure 7:**
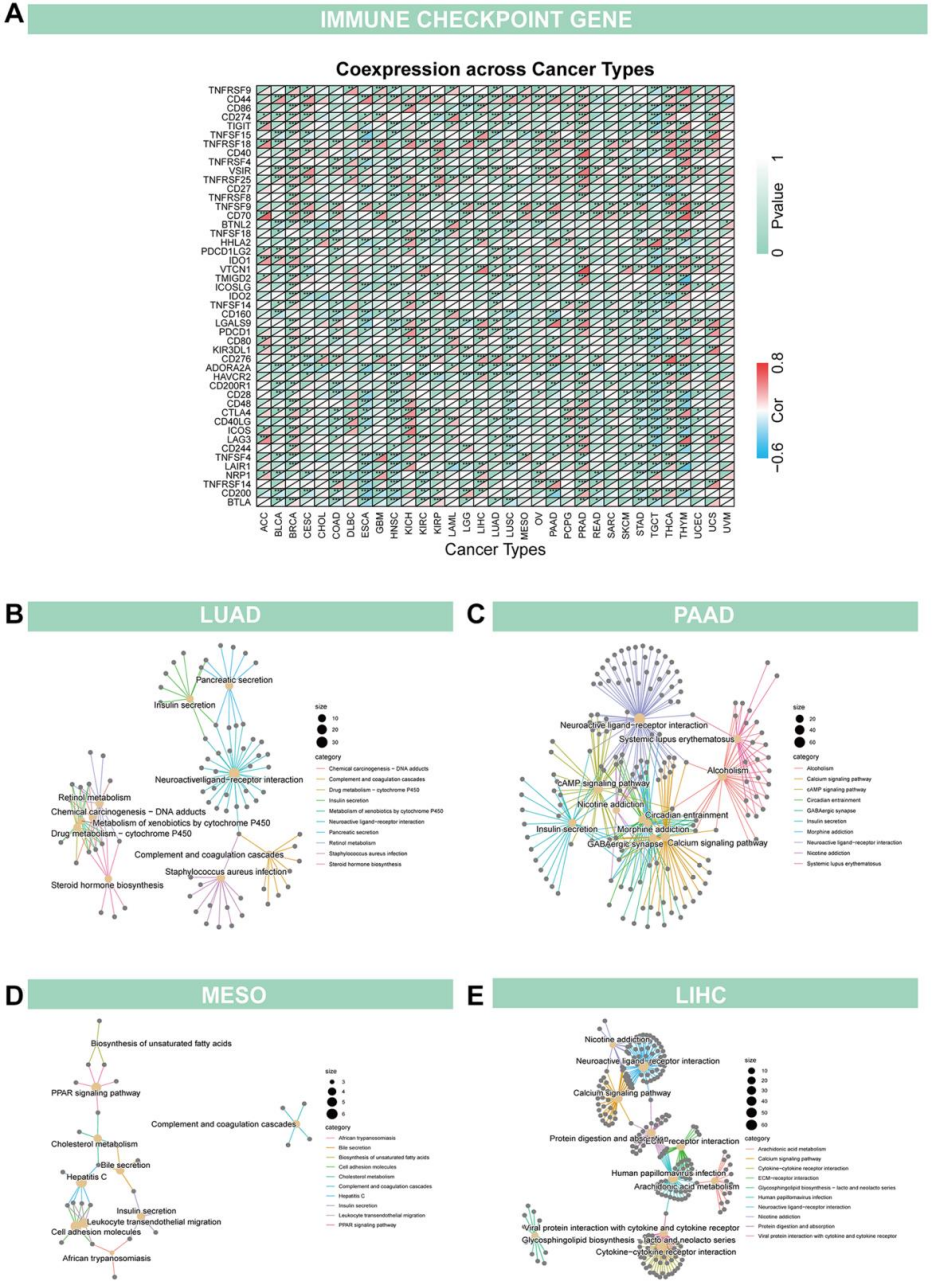
Relationship of GJB3 with immune checkpoint and biological function in cancer (**A**) GJB3 is related to immune checkpoint-related genes in diverse tumors. (**B**–**E**) The KEGG study investigates the biological functions of four cancers, LUAD (**B**), PAAD (**C**), MESO (**D**) and LIHC (**E**).

In addition, we evaluated the relationship between GJB3 levels and immune cell scores in the various cancers via different algorithms. First, we used CIBERSORT to investigate the relationship between GJB3 expression and immune cell infiltration levels (Figure 6A). According to these findings, GJB3 level exhibited a significant correlation with scores of immune infiltration in different types of tumors, particularly with macrophages M0 and dendritic cells. We also quantified six subpopulations of immune cells in the TCGA dataset using the TIMER algorithm. As illustrated in Figure 6B, increased GJB3 expression was associated with different types of immune cells, including T cells CD4, T cells CD8, neutrophils, macrophages, and dendritic cells (DC) in various cancer types. The expression of GJB3 appears to be correlated with the infiltration of multiple immune cells within tumors in certain cancer types such as TGCT, PRAD, and THYM. To be more concise, the presence of GJB3 expression in these cancer types is associated with a higher infiltration of immune cells into the microenvironment of the tumor. The targeting antibody based on immune checkpoint inhibitors (ICI) prevents tumor cells from being attacked by components of the immune system [15]. For a better understanding of the role of GJB3 in predicting the efficacy of ICI therapy, we examined the correlation between the level of GJB3 expression and several immunotherapy predictive biomarkers (Figure 7A), TMB, and MSI. Based on the results, there was a positive correlation between GJB3 expression and TMB in ACC, THYM, STAD, PAAD, and KICH, while a negative correlation was observed in UCEC, SKCM, PRAD, OV, LIHC, and DLBC (as shown in Figure 6C). Further analysis revealed a significant correlation between the expression of GJB3 and MSI across various cancers, as illustrated in Figure 6D. This correlation was particularly notable in BRCA, UCEC, STAD, SKCM, and PRAD. These findings are consistent with previous trends observed in our analyses. Thus, GJB3 may be considered an effective biomarker for assessing the efficacy of immunotherapy in these specific cancer types.

### Enrichment analysis of GJB3 across cancers

Given the strong correlation between GJB3 and prognosis for LUAD, PAAD, MESO, and LIHC based on K-M curves and Cox analyses, a KEGG investigation was conducted to explore the biological role of GJB3 in these four types of cancers (Figure 7B–7E). The top 10 enrichment results were visualized, excluding results at *P* > 0.05. Our findings indicate that, GJB3 may play an important role in various types of cancer, especially in signal transduction-related pathways, as well as in a variety of metabolic processes.

### GJB3 knockdown inhibits proliferation and migration of diverse cancer cells *in vitro*

**Figure 8:**
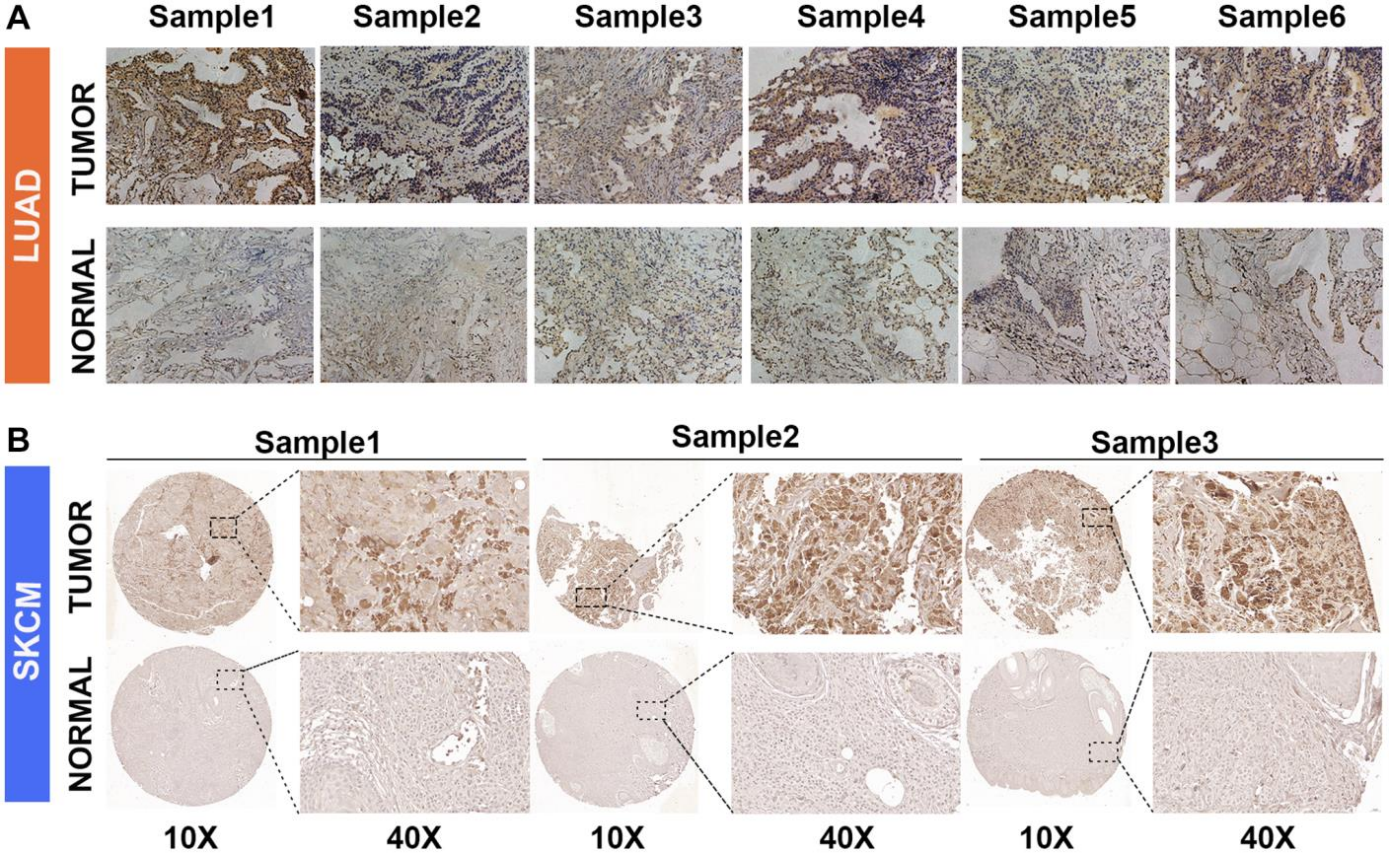
**GJB3 expression in non-tumor and tumor tissues based on immunohistochemistry.** (**A**, **B**) The expression of GJB3 in LUAD (**A**) and SKCM (**B**) was higher in cancer tissues than in non-cancer tissues.

**Figure 9:**
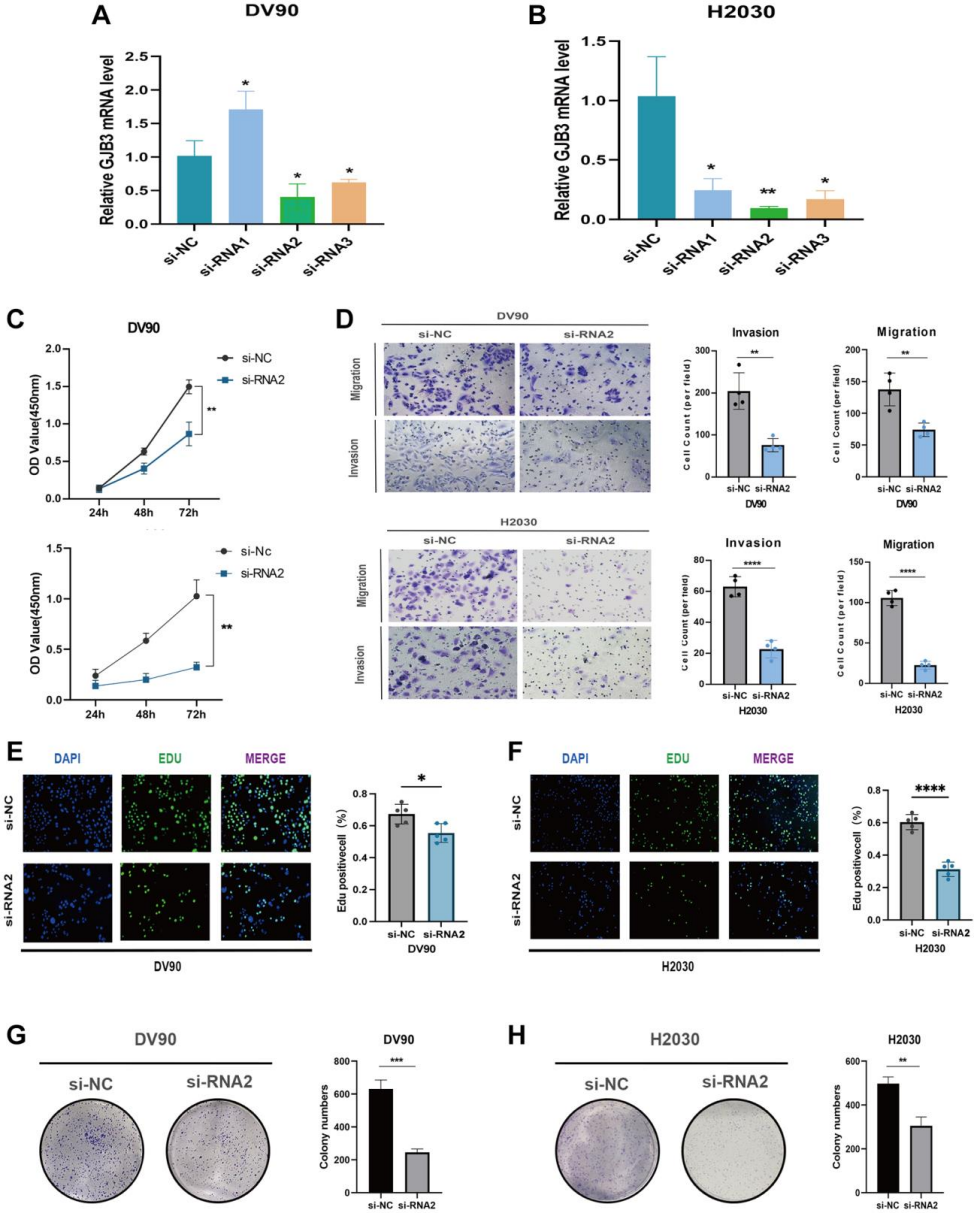
**GJB3 knockdown inhibits the growth and migration of LUAD cells *in vitro*.** (**A**, **B**) Verification of knockdown efficiency of GJB3 in DV90 (**A**) and H2030 (**B**) cell lines. (**C**–**H**) The biological functions of GJB3 in LUAD cell lines were verified by CCK-8 (**C**), transwell (**D**), EDU (**E**, **F**), and colony formation (**G**, **H**). (^*^P < 0.05; ^**^P < 0.01; ^***^P < 0.001; ^****^P < 0.0001).

We found that GJB3 is differentially expressed across several cancer types, acting as an indicator of poor prognosis based on the comprehensive analysis described above. Our immunohistochemical study confirmed this finding, demonstrating increased levels of GJB3 expression in LUAD and SKCM cancer tissues compared with non-cancerous tissues (Figure 8A, 8B). Given the pronounced association of GJB3 with the prognosis in LUAD, MESO, and PAAD, we strategically chose these specific cancer cell lines for our *in vitro* studies to comprehensively investigate its underlying biological functions. Then, we ordered targeted GJB3 small interference RNA, and we used specific GJB3-targeting siRNAs to knockdown the level of GJB3 expression in the lung cancer cells (H2030 and DV90) (Figure 9A, 9B). Based on a comparison of siRNA knockdown efficiency, the second sequence was selected for further experiments. To determine whether high expression of GJB3 affects the growth of LUAD cells, CCK-8 (Figure 9C), the EDU (Figure 9E, 9F), and colony formation assay (Figure 9G, 9H) were conducted. Next, metastatic ability of different levels of GJB3 in cells was assessed using cellular migration and invasion experiments (Figure 9D). Based on the results of our study, we discovered that GJB3 knockdown significantly reduced the growth of both DV90 and H2030 cells. Moreover, the number of DV90 and H2030 cells that migrated and invaded following the GJB3 knockdown was also significantly lower than the control group.

**Figure 10:**
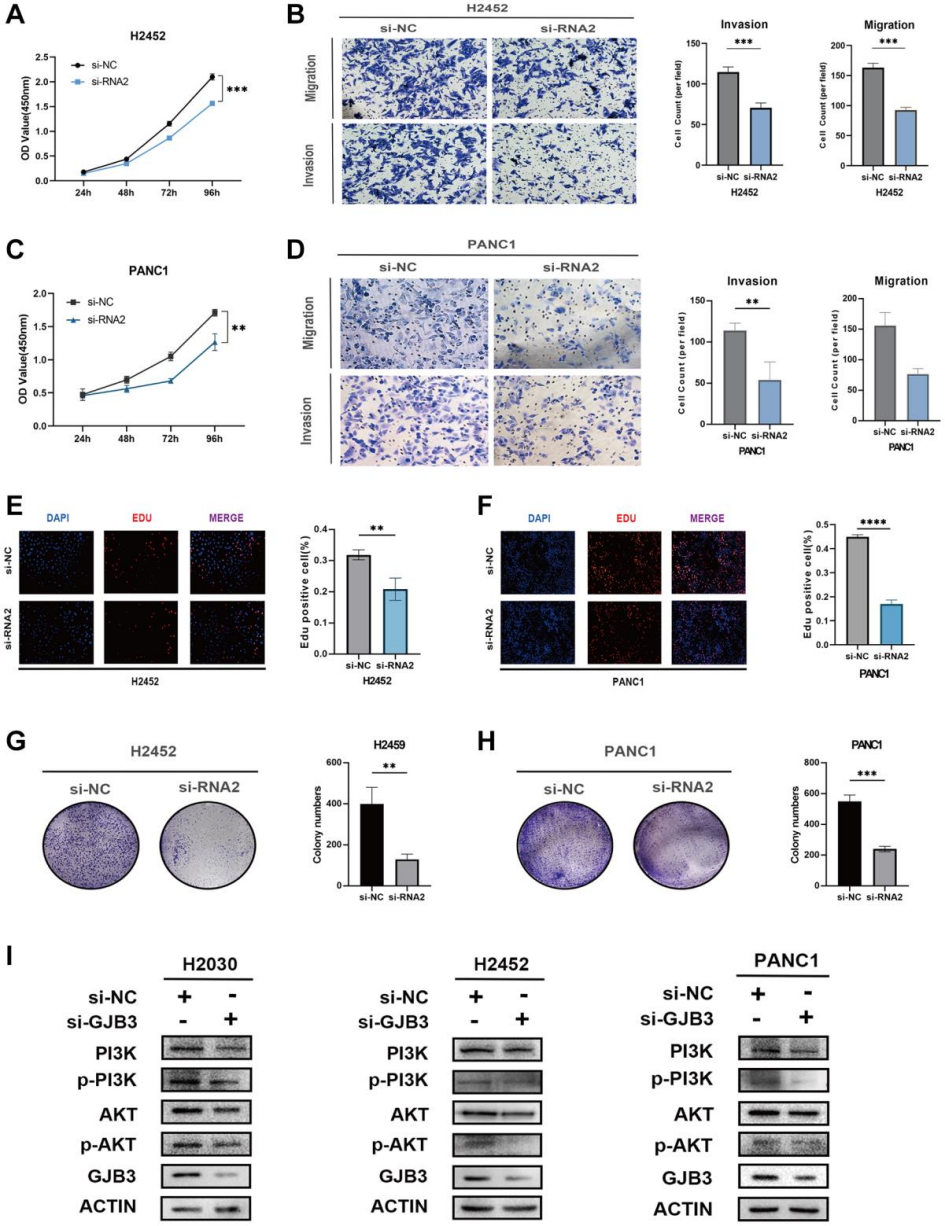
**GJB3 knockdown inhibits the growth and migration and the PI3K/AKT pathway of PAAD and MESO cells.** (**A**–**H**) The biological functions of GJB3 in PANC1 and H2452 cells were verified by CCK-8 (**A**, **C**), transwell (**B**, **D**), EDU (**E**, **F**), and colony formation (**G**, **H**). (**I**) GJB3 knockdown suppressed the PI3K/AKT signaling pathway in H2030, PANC1 and H2452 cells. (^*^P < 0.05; ^**^P < 0.01; ^***^P < 0.001; ^****^P < 0.0001).

Extending this approach, we replicated these experiments in PAAD and MESO cancer cell lines (PANC1 and H2452). The outcomes mirrored those observed in LUAD cell lines, with GJB3 knockdown significantly impeding both proliferation and migration in these cancer types as well (Figure 10A–10H), reinforcing the potential universal role of GJB3 in tumor progression across various cancers.

### GJB3 knockdown suppresses the PI3K/AKT signaling pathway in various cancer cells

The PI3K/AKT pathway, an intracellular signaling cascade, plays a crucial role in cellular responses to external signals, promoting metabolism, proliferation, cell survival, growth, and angiogenesis. Notably, in the cancer field, PI3K/AKT pathway is integral to the regulation of energy metabolism. Previous KEGG analysis results have indicated a significant association between GJB3 and the metabolic processes in various cancers. Therefore, we hypothesized that GJB3 may exert its influence in multiple cancer types through the modulation of the PI3K/AKT pathway. To better understand the functional mechanism of GJB3, we assessed its impact on the PI3K/AKT pathway in LUAD, PAAD, and MESO cell lines using Western blot analysis. Following the knockdown of GJB3, we observed changes in PI3K/AKT pathway activity in H2030, PANC1, and H2452. Specifically, the phosphorylation levels of key PI3K/AKT signaling intermediates, including phosphorylated PI3K (p-PI3K) and AKT (p-AKT), were significantly suppressed upon GJB3 knockdown (Figure 10I).

These findings not only corroborate the pivotal role of GJB3 in modulating the PI3K/AKT pathway but also provide critical insights into its mechanism of action across different cancer cell types. This study further underscores the significance of GJB3 as a potential target for cancer therapy, particularly in strategies involving the PI3K/AKT signaling pathway.

### GJB3 expression and potential therapeutic benefits

**Figure 11:**
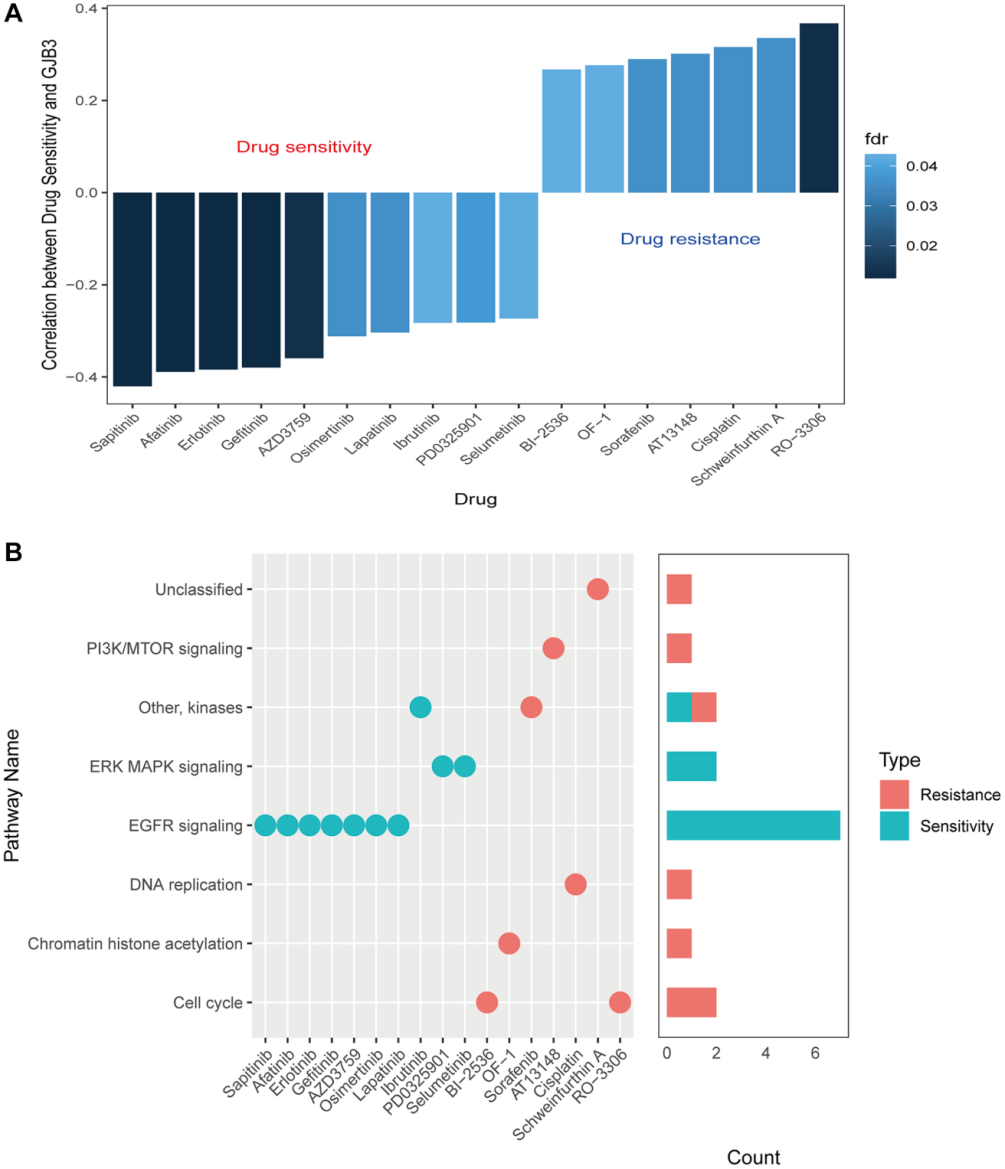
**Drug sensitivity and expression of GJB3.** (**A**) A graph showing the relationship between GJB3 expression and drug sensitivity as calculated by the Spearman algorithm. In each column, the color indicates the fdr, while the height indicates the correlation coefficient. (**B**) This graph visualizes the signaling pathways targeted by GJB3-sensitive and GJB3-resistant drugs. Right: a bar plot representing the number of drugs targeting each pathway.

Given the prominent prognostic role of GJB3 in lung adenocarcinoma, we next explored the possible implications of GJB3 expression levels in response to various medicines to identify new avenues of personalized therapy. Our study investigated the correlation between GJB3 expression and the drug response in multiple lung adenocarcinoma cell lines. Utilizing the GDSC database for reference, our analysis revealed that among the 17 evaluated drugs, their cell-killing efficacy varied significantly in correlation with GJB3 expression levels. Specifically, the sensitivity of 10 drugs positively correlated with increased GJB3 expression, while 7 drugs showed a negative correlation. (Figure 11A). Drugs with increased effectiveness in the presence of higher GJB3 levels are classified as ‘GJB3-sensitive drugs’. Conversely, those showing decreased efficacy with higher GJB3 expression are termed ‘GJB3-resistant drugs’. The GJB3-sensitive drugs include the EGFR inhibitor, Sapitinib, and the selective Bruton’s tyrosine kinase (Btk) inhibitor, Ibrutinib, as well as the selective and non-ATP-competitive MEK inhibitor, PD0325901, which predominantly target the EGFR and ERK-MAPK signaling pathways (Figure 11B). Alternatively, there are GJB3-resistant drugs, such as RO-3316, a cell cycle blocker, and chemotherapy drug, Cisplatin, which primarily target the PI3K/MTOR and cell cycle signaling pathways (Figure 11B). These results indicate that GJB3 holds promise as a biomarker for lung adenocarcinoma therapy, paving the way for the development of personalized treatment strategies that target specific drugs in cancer therapy.

## DISCUSSION

Connexins at gap junctions mediate the communication between cells via a direct exchange of intercellular small molecules (1 kDa). There are over 20 connexin genes in the human genome [16]. Connexins have been shown to be widely distributed among normal-normal cells, cancer-cancer cells, and cancer-normal cells. Moreover, tumor-stroma cell interactions impact cancer progression and therapy responses, and the exchange of information by cancer cells via connexins is associated with tumor progression [17, 18]. In recent years, connexins have been recognized for their role in tumor evolution, as well as their potential role as pan-oncogenes which play unique roles in the origin and progression of cancerous diseases.

The protein GJB3, also called Connexin 31, belongs to a group of proteins known as connexins. These proteins play a crucial role in forming channels and junctions that enable communication between cells. In the past, connexins have been reported to be associated with cancer grade and stage [19], with abnormal expression and localization of connexins being associated with cancer initiation and progression. For example, Connexin 43-mediated gap junctions facilitate short-range fibroblast-lung cancer cell interactions, resulting in chemoresistance [17, 20–22]. As a potential drug target for the failure of chemotherapy, Connexin 32 internalization by USP14 inhibition modulates cisplatin resistance in ovarian cancer cells [22]; however, little attention has been paid to the role of GJB3 in cancer, especially from a pan-cancer perspective. For the first time, our study used multiple databases to uncover the role of GJB3 in cancer and its impact on the immune microenvironment, verified its role in lung adenocarcinoma, and explored its clinical effectiveness in guiding drug treatment. The findings of this study suggest that GJB3 is widely distributed throughout most cancer tissues, and it is commonly expressed at high levels compared with that of normal tissues. Moreover, GJB3 mRNA expression demonstrated significant correlation with the clinicopathological stages of COAD, KIRC, ESCA, BRCA, THCA, READ, LUAD, and KIRP. Additionally, both Cox analysis and K-M curves indicated a relationship between the level of GJB3 expression and OS, PFI, and DSS in various cancer types. It is clear from these findings that GJB3 plays a significant role in various cancers, as predicted by our hypothesis. Thus, GJB3 dysregulation may affect the progression of various cancers.

Tumor-infiltrating immune cells (TIICs) are cancer cells that invade immune cells and play an important role in tumorigenesis. The heterogeneity of TMEs may contribute to the ineffectiveness of various treatments for some patients. Our study found that GJB3 expression was associated with immune cell infiltration in a variety of tumors, particularly TGCA, PRAD, and THYM. Furthermore, over the past few decades, human immunotherapies have yielded novel therapeutic strategies for patients with cancer and have profoundly altered the landscape of oncology; however, not all patients can benefit from immunotherapy and sustain a long-term clinical response. The checkpoint inhibitor class of immunotherapy has been the most extensively researched to date [23]. T cells are capable of distinguishing tumor cells from normal cells. In addition, cancer cells are more likely to be recognized if they contain immunogenic neoantigens. Historically, since neo-antigens have been produced by mutations, the greater the number of mutations (the higher the TMB), the higher the chance that some neo-antigens presented by Major histocompatibility complex (MHC) proteins will be immunogenic, enabling T cell recognition and cancer cell eradication [24–26]. Furthermore, cancers harboring a defective mismatch repair (dMMR) mechanism are often hypermutated and accumulate mutations in monomorphic microsatellites (short tandem repeats) that are particularly prone to mismatch errors. This condition is termed MSI [27]. Moreover, high MSI and DNA dMMR can lead to the accumulation of DNA mutations in tumor cells, resulting in the formation of sufficient tumor neoantigens to induce a strong T cell response and tumor immune responses. Hence, the level of MSI, TMB, and immune checkpoint expression is complex and varies according to the type of tumor. As a predictive biomarker for immunotherapy, they play a significant role. Our study examined the relationship between GJB3 and multiple immune checkpoints, including TMB and MSI, and found that TMB and MSI were associated with GJB3 expression. Furthermore, multiple immune checkpoints were significantly correlated with changes in GJB3, particularly in KICH, PRAD, and THYM. According to our findings, GJB3 participates in the regulation of the immune system, and its expression may be a predictive marker of tumor immunogenicity and therapeutic response.

Due to their high permeability to small molecules and macromolecules, GJB3 channels are highly attractive targets for delivering drugs directly into the cell cytoplasm. Based on the GDSC database analysis, we observed an association between the level of GJB3 expression and the sensitivity to specific drugs. These results suggest that the level of GJB3 expression could be used to predict the response of lung adenocarcinoma cells to certain drugs and, thus, may be a useful prognostic biomarker. In the future, this could lead to improved survival outcomes for cancer patients.

The present study has extensively examined the role of GJB3 in pan-cancer from a bioinformatics perspective; however, there are some limitations. Although it is clear that abnormal GJB3 expression correlates with immune cell and prognosis in cancers, it remains uncertain whether GJB3 directly affects patient survival through the immune response. It remains necessary to obtain more direct evidence of the role of GJB3 in influencing patient survival. Moreover, while these findings have opened up new areas for future studies, additional clinical and experimental data are required to supplement and validate the potential biological processes and molecular mechanisms involved.

## CONCLUSIONS

We conducted a comprehensive evaluation of the GJB3 gene expression signature, including its prognostic value and association with immune cell infiltration and cancer-associated pathways across various cancer types. The results of the study demonstrated that GJB3 is abnormally expressed and typically predicts a poor outcome in various cancers, especially in LUAD, PAAD and MESO. Moreover, these results suggest that GJB3 may represent a promising novel immune target in tumors, with a potential regulatory role in the immune response.

## Supplementary Materials

Supplementary Tables
