# Supplementary material for: GJB3: a comprehensive biomarker in pan-cancer prognosis and immunotherapy prediction
Source: Aging (Albany NY). 2024 May 7;16(9):7647–67. doi: 10.18632/aging.205774 (PMC11132019; doi:10.18632/aging.205774)
Supplement: Supplementary Tables [file aging-16-205774-s001.pdf]

SUPPLEMENTARY TABLES

Supplementary Table 1. List of abbreviations of cancer names used in this study.

Cancer abbreviations	Corresponding meanings of caner abbreviations
ACC	Adrenocortical carcinoma
BLCA	Bladder urothelial carcinoma
BRCA	Breast invasive carcinoma
CESC	Cervical squamous cell carcinoma and endocervical adeno carcinoma
CHOL	Cholangiocarcinoma
COAD	Colon adenocarcinoma
READ	Rectum adenocarcinoma esophageal carcinoma
DLBC	Lymphoid neoplasm diffuses large B-cell lymphoma
ESCA	Esophageal carcinoma
GBM	Glioblastoma multiforme
HNSC	Head and neck squamous cell carcinoma
KICH	Kidney chromophobe
KIRC	Kidney renal clear cell carcinoma
KIRP	Kidney renal papillary cell carcinoma
LAML	Acute myeloid leukemia
LGG	Brain lower-grade glioma
LIHC	Liver hepatocellular carcinoma
LUAD	Lung adenocarcinoma
LUSC	Lung squamous cell carcinoma
MESO	Mesothelioma
OV	Ovarian serous cystadenocarcinoma
PAAD	Pancreatic adenocarcinoma
PCPG	Pheochromocytoma and paraganglioma
PRAD	Prostate adenocarcinoma
READ	Rectum adenocarcinoma
SARC	Sarcoma
SKCM	Skin cutaneous melanoma
STAD	Stomach adenocarcinoma
STES	Stomach and esophageal carcinoma
TGCT	Testicular germ cell tumors
THCA	Thyroid carcinoma
THYM	Thymoma
UCEC	Uterine corpus endometrial carcinoma
UVM	Uveal melanoma

Supplementary Table 2. The primer sequences and siRNA sequences used in this study.

GJB3	Forward	CCTCCTCCTATGGACTGCCC
	Reverse	AAGGCCGTGAAGTCTGGGATA
GAPDH	Forward	GGAGCGAGATCCCTCCAAAAT
	Reverse	GGCTGTTGTCATACTTCTCATGG
GJB3 siRNA1	Forward	CAAGCGAAUUAACUAUCUACG
	Reverse	UAGAUAGUUAAUUCGCUUGUU
siRNA2	Forward	CUCUGAGUUCACUAAGUUAUG
	Reverse	UAACUUAGUGAACUCAGAGUG
siRNA3	Forward	CCAACGUCUGCUACGACAACU
	Reverse	UUGUCGUAGCAGACGUUGGUG
